# A new Triassic austrolimulid from Poland presents insight into xiphosurid evolution and palaeobiogeography at the dawn of the Mesozoic

**DOI:** 10.7717/peerj.20950

**Published:** 2026-03-25

**Authors:** Jonatan Audycki, Russell D.C. Bicknell, Grzegorz Niedźwiedzki, Kenneth De Baets

**Affiliations:** 1 Institute of Evolutionary Biology, Faculty of Biology, University of Warsaw, Warsaw, Poland; 2 Division of Paleontology, American Museum of Natural History, New York, United States of America; 3 Palaeoscience Research Centre, School of Environmental & Rural Science, University of New England, Armidale, New South Wales, Australia; 4 Department of Organismal Biology, Evolutionary Biology Center, Uppsala Universitet, Uppsala, Sweden; 5 Polish Geological Institute—National Research Institute, Warsaw, Poland

**Keywords:** *Limulus*, Horseshoe crab, Arthropoda, Buntsandstein, Holy Cross Mountains

## Abstract

Xiphosurids are aquatic chelicerates widely viewed as examples of so-called ‘living fossils’ due to their apparent morphological conservatism and limited diversity since at least the Jurassic. However, earlier representatives were much more diverse and morphologically disparate. Particularly striking are hypertrophied genal spines and reduced thoracetrons of the Triassic austrolimulids, possibly related to their colonization of brackish or freshwater habitats. Here we describe *Polonolimulus zaleziankensis* gen. et sp. nov., a new austrolimulid genus from the Early Triassic of Holy Cross Mountains, Poland. Geometric morphometric analysis positions the new find among the morphologically most ‘extreme’ austrolimulids, extending the geographic range of those forms to Central Europe. A palaeobiogeographic reconstruction of Triassic xiphosurids reveals their surprisingly wide distribution already in Early Triassic, suggesting either an earlier dispersal in the Late Permian or a rapid diversification in the earliest Triassic. The reconstruction of most austrolimulid occurrences within or proximal to the shallow marine areas casts doubts on the hypothesis they inhabited fully freshwater palaeonvironments, which should be reinvestigated in the future. The new material further adds to the growing understanding of xiphosurid diversity and evolution in the early Mesozoic.

## Introduction

Xiphosurids, commonly known as horseshoe crabs, are a group of aquatic chelicerates that have shown very limited morphological change over the past 200 million years (Bicknell et al., 2022a). Such striking morphological conservatism has become an important subject in the broader discussions of evolutionary rates in biology and palaeontology, with horseshoe crabs often serving as a model of a slowly evolving (bradytelic) lineage and the modern forms dubbed ‘living fossils’ or ‘stabilomorphs’ (Simpson, 1944; Barthel, 1974; Fisher, 1984; Kin & Błazejowski, 2014; Dhar et al., 2016). While such limited diversity and morphological disparity may be present in the xiphosurid lineage since the Jurassic, earlier fossil horseshoe crabs evolved strikingly different forms, especially the Carboniferous belinurids and the Triassic austrolimulids (Bicknell & Pates, 2020). Both these groups have been suggested to inhabit non-marine environments, their ‘aberrant’ morphologies, especially the long genal spines, and high diversity (relative to other xiphosurid clades) possibly resulting from adaptive radiations in these new habitats (Lamsdell, 2016; Lamsdell, 2020; Bicknell & Pates, 2020). The Triassic horseshoe crabs are particularly interesting, as it is the time of the second (and last) major increase in their anatomical disparity, stemming from the coexistence of two xiphosurid families with distinct morphologies—the ‘extreme’ austrolimulids, characterized by their often considerably splayed (laterally-oriented), hypertrophied genal spines and/or reduced thoracetrons, and the more ‘standard’ limulids, reminiscent of Recent forms (Bicknell et al., 2019; Bicknell et al., 2022a; Bicknell & Shcherbakov, 2021). The brackish/freshwater habitat suggested for many austrolimulids further separates them from predominantly marine limulids (Lamsdell, 2016; Bicknell & Pates, 2020; Bicknell, Hecker & Heyng, 2021). Investigations of this apparent contrast between the diverse austrolimulids and the morphologically and ecologically conservative limulids could provide insight into the fundamental drivers and constraints influencing horseshoe crab evolution. However, austrolimulid ecology and evolution remains poorly understood, the uncertainties including the exact taxonomic composition of the group, the functional morphology of its most ‘extreme’ representatives and accuracy of palaeohabitat reconstructions (Lamsdell, 2020; Bicknell & Shcherbakov, 2021; Bicknell, Hecker & Heyng, 2021; Lerner & Lucas, 2022; Bicknell et al., 2022a). These issues are exacerbated by the relative scarcity of xiphosurid fossils, highlighting the importance of any novel find (Bicknell et al., 2021b). Here we describe a new fossil horseshoe crab from the Early Triassic of Holy Cross Mountains in southern Poland and assign it to Austrolimulidae Riek, 1955. This new find increases the diversity of the morphologically ‘extreme’ austrolimulids and sheds more light on the evolution and distribution of early Mesozoic xiphosurids.

**Figure 1:**
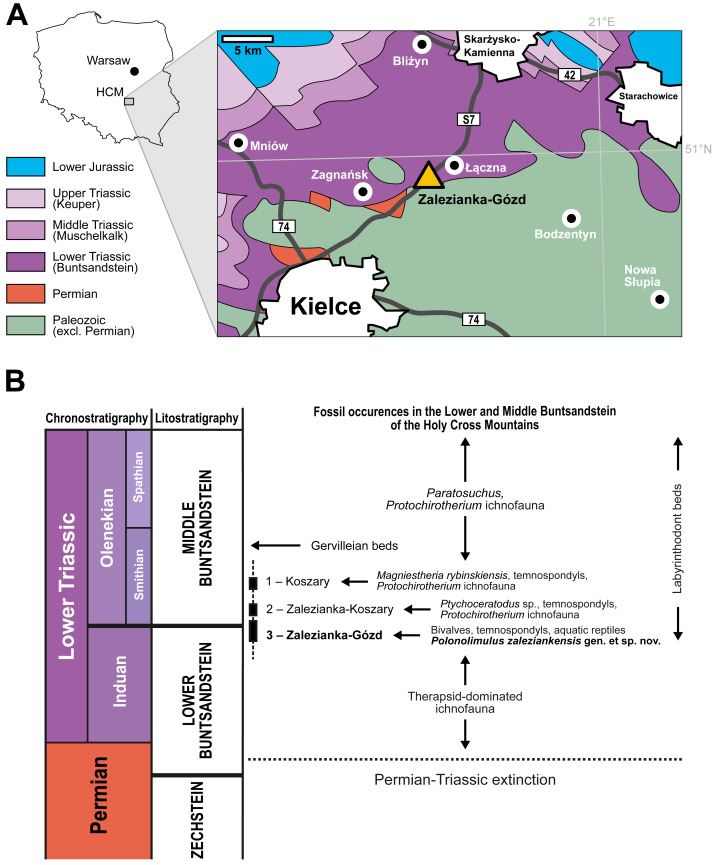
Geological setting and stratigraphy of the Zalezianka-Gózd locality. (A) Location of the Holy Cross Mountains (HCM) on the contour map of Poland and a simplified stratigraphic map of the region encompassing the Zalezianka-Gózd outcrop. The map is based on the 1:1000000 geological map of Poland edited by Dadlez, Marek & Pokorski (2000). (B) Chrono-, lito- and biostratigraphy of the Lower and Middle Buntsandstein in the Holy Cross Mountains with information about the positions of the 1—Koszary, 2—Zalezianka-Koszary and 3—Zalezianka-Gózd sections, with an overview of their respective fossil faunas and ichnofaunas. Lithostratigraphy after Kuleta & Zbroja (2006) and Ptaszyński & Niedźwiedzki (2006).

### Geological setting

**Figure 2:**
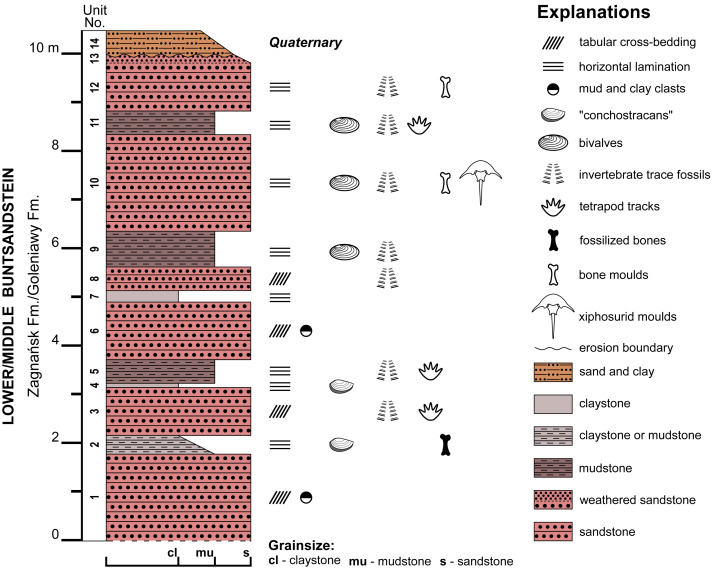
Geological section of the Zalezianka-Gózd locality. The generalized, composite geological section showing the Lower/Middle Buntsandstein deposits correlated to Zagnańsk/Goleniawy formations, exposed in the vicinity of villages Zalezianka and Gózd.

The Zalezianka-Gózd site (also known as Gózd-Występa), discovered in 2005, is a fossiliferous locality that yielded tetrapod bone fragments and tracks, diplostracans (“conchostracans”, clam shrimps), bivalves and invertebrate trace fossils, among others (G Niedźwiedzki, 2025, unpublished data). The site is located at a road-cut along the S7 expressway between Kielce and Skar.zysko-Kamienna, near Łączna, about 12 km northeast of Kielce in the central part of the Holy Cross Mountains Mesozoic margin (Fig. 1A). Other fossiliferous sites in close proximity have also been explored (Skrzycki, Niedźwiedzki & Tałanda, 2018; Fig. 1B). A partially excavated stratigraphic profile was well visible at the Zalezianka-Gózd site between 2007–2010 and included a composite ∼10 m section of red to reddish-brown sandstones and mudstones with claystone intercalations. The sequence was uncovered during road construction and a Buntsandstein outcrop over 30 m long was available for observation from 2007 to 2010. Lithofacies and measured palaeocurrent directions indicate that the area was drained by rivers flowing north, north-east, and north-west. Five facies types (1–medium-grained tabular-cross laminated sandstones with mud and clay clasts; 2–fine-grained horizontal laminated sandstones; 3–mudstones with indistinct horizontal lamination; 4–mudstone/claystone heteroliths; 5–massive claystones with horizontal lamination) have been identified in the section based on field observations of lithologies, sedimentary structures, and textures (Fig. 2). Sedimentological observations indicate medium-energy, braided river depositional environments, periodically transporting rather poorly sorted sandy material (lower part of the section). The sequence also includes sediments from stagnant reservoirs (upper part of the profile) that are likely off-channel deposits from shallow, periodically supplied floodplain reservoirs. Investigated xiphosurid fossils have been found in this type of deposits (Fig. 2). This part of the sequence yielded numerous fossils suggesting the brackish or marginal marine character of the fauna inhabiting this area (marine bivalves, trematosaurid temnospondyls, aquatic reptiles). This indicates a connection between northern margin of the Holy Cross Mountains area and the Boreal Sea basin in the north or margin of the Tethys basin in the south. More detailed palaeogeographical interpretations and proposed correlations will be presented in a separate publication. It is worth noting that apparent marine influences in the Polish Buntsandstein were previously recognized in several stratigraphical intervals: in the Lower Buntsandstein (Pieńkowski, 1991; Szulc, 2019), in the lower part of the Middle Buntsandstein (Becker, Fijałkowska-Mader & Jasionowski, 2020), and at the end of Buntsandstein sedimentation, when marine transgression culminated in the formation of the so-called Muschelkalk basin (Szulc, 2000).

This interval can be correlated to the upper Lower Buntsandstein or lower Middle Buntsandstein of the Holy Cross Mountains based on these basic lithostratigraphic observations. However, the precise placement of this sequence in the local lithostratigraphic scheme presents certain problems, which result from the poor knowledge of the lateral variability of the Buntsandstein in this stratigraphic interval. The Zalezianka-Gózd section is located below the Middle Buntsandstein interval with characteristic faunas: 1–Koszary and 2–Zalezianka-Koszary (= Zalezianka site in Skrzycki, Niedźwiedzki & Tałanda, 2018) sections (Fig. 1B). The first site (Koszary) contains a typical Olenekian tetrapod ichnofauna (cf. *Synaptichnium* isp., *Protochirotherium* isp., *Rhynchosauroides* isp., and cf. *Prorotodactylus* isp. tracks), and “conchostracan” fossils, which include *Magniestheria rybinskiensis* (Novozhilov, 1946; Niedźwiedzki, Brusatte & Butler, 2013). The Zalezianka-Koszary site yielded small and fragmentary bones of temnospondyl amphibians, natural cast of the lower tooth plate of dipnoan *Ptychoceratodus* sp., plant roots, and swimming fishes traces, tetrapod tracks and invertebrate trace fossils such as arthropod trails and cf. *Planolites* isp. (Skrzycka et al., 2014; Skrzycki, Niedźwiedzki & Tałanda, 2018). Although the tetrapod tracks from Zalezianka-Koszary site have not yet been formally described, the collected material suggests presence of the tetrapod ichnoassemblage with *Protochirotherium* and *Rynchosauroides* tracks.

The presence of *Magniestheria rybinskiensis* Novozhilov, 1946 indicates an early Olenekian age (Smithian substage) for the Koszary section (Niedźwiedzki, Brusatte & Butler, 2013). The paleontological content of the Zalezianka-Koszary site located below the Koszary site suggests its earliest Olenekian or latest Induan age (Skrzycki, Niedźwiedzki & Tałanda, 2018). In contrast to the Zalezianka-Koszary site, numerous “conchostracans” were found in the Zalezianka-Gózd site, but could not be identified in detail due to poor preservation. The age of this part of the section therefore requires further biostratigraphic studies. The presence of numerous temnospondyl bones may suggest that such fossiliferous sequences in this region are so-called “Labyrinthodont beds” (Senkowiczowa, 1970; Kuleta & Zbroja, 2006).

The first finds of bivalve fossils in the Zalezianka-Gózd section (assigned to Bakevelliidae King, 1850) suggest similarities to fauna described from the so-called “Gervilleian beds”, an informal lithostratigraphic unit from Middle Buntsandstein interpreted as lacustrine or marine-brackish deposits (Senkowiczowa, 1970; Kuleta & Zbroja, 2006). The preliminary taxonomical determinations of Zalezianka-Gózd fossils demonstrates that they represents another, stratigraphically older fauna (Fig. 1B), containing at least three bivalve genera, trematosaurid temnospondyls aquatic reptiles (an on-going study), and the xiphosurid described here.

The Zalezianka-Gózd section can be locally correlated to the Lower Buntsandstein (Zagnańsk Formation) or Middle Buntsandstein units (of Goleniawy/Zagnańsk formations) from the western and central part of the Holy Cross Mountains based on lithostratigraphical features (Kuleta & Zbroja, 2006; Ptaszyński & Niedźwiedzki, 2006).

## Materials & Methods

### Investigated material

**Figure 3:**
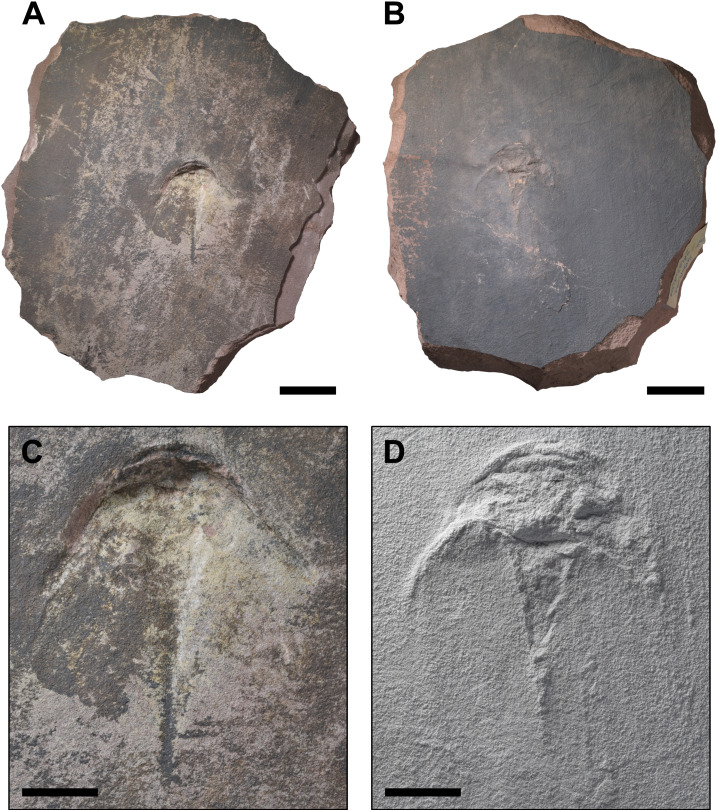
The rock slab bearing Muz. PGI 1808.II.10 and another xiphosurid fossil. (A) The upper surface of the rock slab with Muz. PGI 1808.II.10 preserved as a concave natural mould. (B) The lower surface of the rock slab with the uncertain convex horseshoe crab-shaped structure, considered a possible trace fossil (see ‘Remarks’ for further discussion). (C) Close-up of Muz. PGI 1808.II.10 (D) Close-up of the convex structure on the lower surface coated with ammonium chloride sublimate. Scale bars: (A, B) 50 mm, (C, D) 20 mm. Photo credit: Jonatan Audycki.

The examined specimens were collected in 2008 by Grzegorz Niedźwiedzki from a temporary Buntsandstein outcrop associated with construction of the S7 expressway near the village of Zalezianka, northeast of Kielce in the Świętokrzyskie (Holy Cross) Voivodeship in southern Poland (50°58′56″N, 20°45′06″E). Two xiphosurid fossils were found preserved as concave impressions in a very fine sandstone, but only one could be collected (Fig. 3), while the second was cast using gypsum. The collected rock slab bearing concave impression also has a convex horseshoe crab-like fossil on the lower surface (Figs. 3B, 3D; see ‘Remarks’). The specimens are housed in the Buntsandstein collection in the Polish Geological Institute-National Research Institute, Warsaw, Poland under the numbers Muz. PGI 1808.II.10 and 11. The convex latex peel of Muz. PGI 1808.II.10 is housed at the Faculty of Biology, University of Warsaw in Warsaw, Poland under number gz4142. The specimens are preserved as natural moulds (external ‘imprints’) of original carapaces, which were buried and dissolved after early lithification of sand material. This is the typical state of preservation of organic remains in this site (Fig. 2), and more generally of the Buntsandstein fossils in the region, with both vertebrate skeletal fragments (scales, teeth or bones) and invertebrate remains (bivalves, gastropods or arthropods) represented by secondarily filled or unfilled external moulds preserved in the silicified sandstone matrix (*e.g.*, Skrzycki, Niedźwiedzki & Tałanda, 2018). Only one bed in this section yielded a vertebrate fossil with fossilized original bone tissue (Fig. 2).

Due to the type of their preservation, the examined specimens are rather poorly preserved, with many anatomical structures deformed or effaced by the taphonomic processes acting on the buried remains during different stages of diagenesis. Nevertheless, both specimens display features and morphological details supporting the interpretation of studied fossils as natural moulds of xiphosurid carapaces. These include the well-defined boundaries of anatomical elements, including genal spines; the distinct demarcation of cephalothorax and thoracetron; and the presence of fine structures such as thoracetronic flange or terminal thoracetronic projections (see ‘Description’). It is improbable that such features would be so well preserved, or even at all discernible, if the specimens were not body fossils but rather resting traces. Additionally, it is worth noting that Muz. PGI 1808.II.10 represents an external mould of the dorsal carapace surface, as evidenced by the conspicuous thoracetronic flange. In the case of trace fossil, the ventral body surface would be expected. The cooccurrence of xiphosurid fossils with similarly preserved bivalves and tetrapod bones (Fig. 2) also supports the interpretation of investigated specimens as natural moulds of carapaces.

### Imaging and description

**Figure 4:**
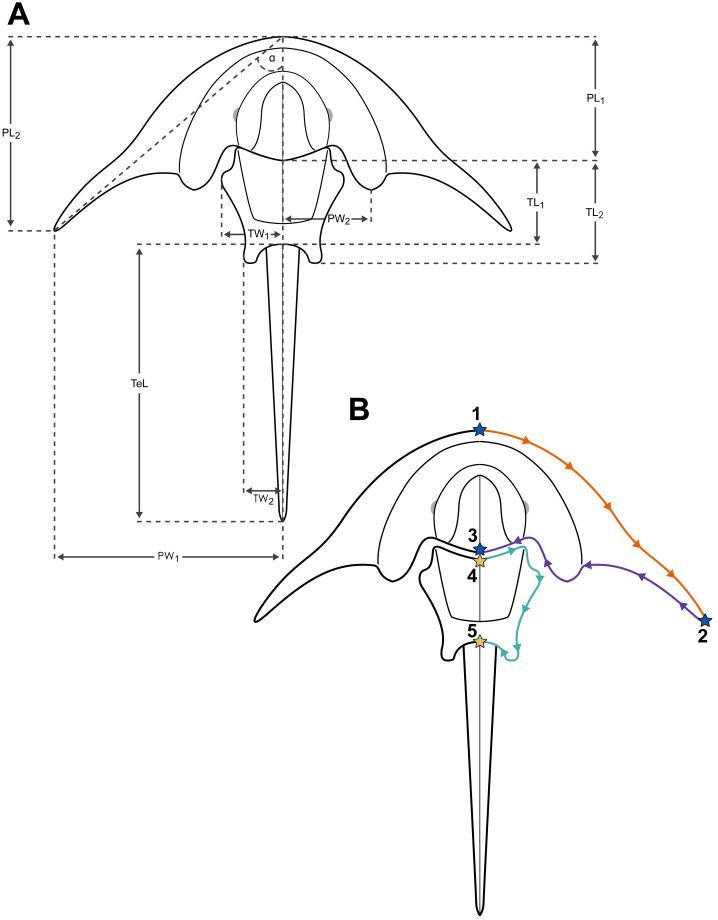
Measuring and landmarking schemes employed in the present study. (A) Diagram of basic measurements performed to augment the description of examined material: α, genal splay *sensu* Lerner, Lucas & Lockley (2017); PL_1_, prosomal length 1 (at midline); PL_2_, prosomal length 2 (at level of genal spine tips); PW_1_, prosomal width 1 (from midline to genal spine tip); PW_2_, prosomal width 2 (from midline to the posterior prosomal lobe terminus); TeL, telson length; TL_1_, thoracetron length 1 (at midline); TL_2_, thoracetron length 2 (to the blunt projections forming ‘swallowtail’); TW_1_, thoracetron width 1 (from midline to anterolateral thoracetronic lobe tip); TW_2_, thoracetron width 2 (from midline to maximum lateral extent of posterior thoracetronic projections). (B) Diagram displaying the fixed landmarks and curves (subsequently subsampled for semi-landmarks) placed on a photograph of each examined specimen. Note that for the PCA variant 2, which used prosomal data only, fixed landmarks 4 and 5 (marked with yellow stars) as well as the thoracetronic curve (teal coloured) were removed from the dataset.

Specimens were photographed using a Nikon D5300 camera with a Sigma 24–35 mm f/2 DG HSM ART and Tamron SP 60 mm F/2 Di II lenses under standard and low-angle LED light. To better visualize and highlight all features, a latex cast of Muz. PGI 1808.II.10 was made and coated with ammonium chloride sublimate. This convex peel, gz4142, was then photographed with a Keyence VHX-7000 digital microscope using the ‘Serial recording → 3D Image Stitching’ function under a normal and low-angle halogen light. The lower surface of the rock slab bearing Muz. PGI 1808.II.10 was also coated with ammonium chloride sublimate to better display the possible trace fossil (Fig. 3D). Select images were converted to grayscale using GNU Image Manipulation Program (GIMP) 2.10.38. A virtual 3D model of the whole rock slab bearing Muz. PGI 1808.II.10 was created using photogrammetry with the RealityCapture Version 1.5.1.118081 RC software. The 3D model and high resolution images of gz4142 are available at the OSF repository: https://doi.org/10.17605/OSF.IO/A4M8F. Morphological terminology used for description follows Bicknell et al. (2021a) with select terms from Lerner, Lucas & Lockley (2017), Bicknell & Shcherbakov (2021), and Lamsdell & Ocon (2025) as well as directional terms from Harrington (1959). Note that while the correct derivative of Xiphosurida is ‘xiphosuridan’, it is very rarely used (*e.g.*, Pazinato et al., 2022) and thus ‘xiphosurid’ was used throughout the text so as to be consistent with the existing literature and for the sake of simplicity. Measurements of the specimens were gathered digitally with ImageJ 1.54 g using photographs of casts (see Fig. 4A for a diagram of performed measurements).

### Geometric morphometry

A 2D geometric morphometric analysis was performed to quantitatively investigate the position of the new taxon in morphospace of known Triassic horseshoe crabs. This approach employs landmark and semi-landmark data (relative positions of defined morphological features, and equally spaced points subsampled from outlines, respectively) collected from all examined specimens to analyse and visualise variation in their shape, usually with the use of Procrustes-fitting and principal component analysis (Hammer & Harper, 2024). Specimen Muz. PGI 1808.II.10 and specimens of *Limulitella tejraensis* Błażejowski et al., 2017 from ZPAL V.46 collection were available directly in Warsaw and thus new high-resolution photographs were produced; for other specimens available photographs were gathered from existing literature (see Table S1 for the complete list of specimens, taxonomic assignment, and sources). Two variants of the analysis were carried out: variant 1 involving both prosomal and thoracetron data, and variant 2 with prosomal data only. Variant 1 included 12 specimens from 11 recognized species for which 5 fixed landmarks and 3 curves were placed, while variant 2 included 19 specimens from 15 recognized species for which 3 fixed landmarks and 2 curves were placed (Fig. 4B). 20 semi-landmarks were subsampled from each curve. The right half of the specimen was always used, regardless of the type of preservation (*e.g.*, natural mould, part/counterpart, or cast of the original fossil). When the right side was less suitable for landmarking, the photographs were mirrored in GIMP to use the left side (Table S1). As the examined horseshoe crab fossils included both three-dimensionally preserved and completely flattened specimens, it is possible some minor taphonomic ‘noise’ was introduced into the analysis. However, such taphonomic differences in horseshoe crab morphometry analysis are minimal and the true biological signal generated by the shape disparity is the most prominent (Bicknell et al., 2019; Bicknell et al., 2022a). Furthermore, due to the scarcity of Triassic xiphosurid fossils excluding additional specimens would result in a significant loss of important morphometric data.

Fossil images were digitized using the ‘StereoMorph’ R package version 1.6.7 (Olsen & Westneat, 2015; Olsen & Haber, 2022). The geometric morphometric analysis was conducted with ‘geomorph’ R package version 4.0.10 (Baken et al., 2021; Adams et al., 2025). Imported landmark and semi-landmark data was subjected to the generalized procrustes analysis (GPA) to standardize the size, orientation and position of the landmark sets from different specimens and enable subsequent examination of shape differences. The semi-landmarks were slid based on minimizing bending energy (the default setting for GPA in ‘geomorph’) to optimize their position and Procrustes aligned specimens were projected into tangent space. A principal components analysis (PCA) was then performed on the Procrustes-fitted data, with results plotted for the two first principal components. For clarity, the PCA coordinates of the examined specimens listed in the ‘Results’ section were rounded to three decimal places. For both variants of the analysis, the convex hulls for the two Triassic horseshoe crab families: Austrolimulidae and Limulidae were plotted in two alternative ways, to account for the contentious affinity of the genus *Limulitella* (Størmer, 1952) and show how the family-level morphological disparity is plotted with *Limulitella* included in either family (*e.g.*, Lamsdell, 2020; Bicknell & Shcherbakov, 2021; Bicknell, Hecker & Heyng, 2021). It is important to note that we used previously defined family-level designations, and have not conducted a phylogenetic analysis herein. Specimen photos, digitized landmark data and complete code used to conduct the analysis is available at OSF: https://doi.org/10.17605/OSF.IO/ZAQK9.

### Palaeobiogeography

To establish the geographic position of the new specimens relative to other Triassic xiphosurid fossils, a palaeobiogeographic reconstruction of the Triassic xiphosurids, *i.e.,* the mapping of reconstructed palaecoordinates of the fossil occurrences on the reconstructed palaeogeographic map of the world in the Triassic, was performed using the ‘rgplates’ R package version 0.6.0 (Müller et al., 2018; Kocsis et al., 2024). The reconstruction involved almost all Triassic localities that yielded previously published xiphosurid body fossils, with a single exception of the site that produced holotype of *Limulitella liasokeuperinus* (Braun, 1860), which has since been lost and is therefore unavailable for further study (Table S2). The palaeogeographic reconstruction used model and shapefiles of Cao et al. (2017), which include the estimated extents of landmasses, shallow seas and mountain ranges, and the rotation model of Matthews et al. (2016). The same model was also used to reconstruct the palaeocoordinates of fossil occurrences. Since a single map was necessary to suitably present the paleobiogeographic distribution of Triassic horseshoe crabs, the reconstruction was performed for 245 Ma, corresponding to Anisian, Middle Triassic (Cohen et al., 2013; updated). As the position of continents and extent of shallow seas gradually changed during the Triassic, this reconstruction does not provide a fully accurate view of the global palaeogeography for each individual occurrence, being rather a broad overview of Triassic horseshoe crab distributions. The reconstructed data was processed and subsequently plotted using the ‘sf’ and ‘ggplot2’ R packages (Wickham, 2016; Pebesma, 2018; Pebesma & Bivand, 2023).

Both the geometric morphometric analysis and the palaeobiogeographic reconstruction were performed in the R environment using R 4.4.2 for Windows with RStudio 2024.12.0+467. For the complete code and shapefiles used for the palaeobiological reconstruction see Data S3. For the purposes of formatting, optimizing the layout of presented information and standardizing the relevant symbols, the figures were further customized with Inkscape and GIMP.

The electronic version of this article in Portable Document Format (PDF) will represent a published work according to the International Commission on Zoological Nomenclature (ICZN), and hence the new names contained in the electronic version are effectively published under that Code from the electronic edition alone. This published work and the nomenclatural acts it contains have been registered in ZooBank, the online registration system for the ICZN. The ZooBank LSIDs (Life Science Identifiers) can be resolved and the associated information viewed through any standard web browser by appending the LSID to the prefix http://zoobank.org/. The LSID for this publication is: urn:lsid:zoobank.org:pub:8CE309E7-0B35-493B-89E4-0D16FB7FFDD5. The online version of this work is archived and available from the following digital repositories: PeerJ, PubMed Central SCIE and CLOCKSS.

## Results

### Systematic palaeontology

**Table:** 

Phylum ARTHROPODA Gravenhorst, 1843
Subphylum CHELICERATA Heymons, 1901
Class XIPHOSURA Latreille, 1802
Order XIPHOSURIDA Latreille, 1802
Suborder LIMULINA Richter & Richter, 1929
Superfamily LIMULOIDEA Zittel, 1885
Family AUSTROLIMULIDAE Riek, 1955

**Table:** 

Genus *Polonolimulus* nov.

**Etymology:** Derived from the combination of *Polonia*, the Latin name for Poland, and *Limulus*, the generic name of the modern American horseshoe crab.

**Type and only species:** *P. zaleziankensis,* gen. et sp. nov.

**Diagnosis:** As for the type species.

**Occurrence:** Only known from the type locality.

**Table:** 

*Polonolimulus zaleziankensis*, gen. et sp. nov.

**Etymology:** Named after the village Zalezianka within the Holy Cross Mountains where the type locality is situated.

**Figure 5:**
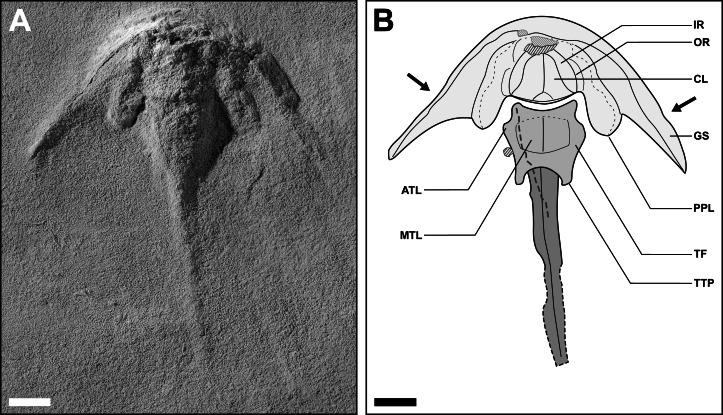
Holotype of *Polonolimulus zaleziankensis* gen. et sp. nov. (latex peel gz4142 of holotype Muz. PGI 1808.II.10). Specimen visible in dorsal view. (A) Latex cast of holotype, gz4142, coated with ammonium chloride sublimate; light from top-left. (B) Interpretative drawing of gz4142 highlighting main features; arrows show the position of genal spine kink. Acronyms: ATL, anterolateral thoracetronic lobe; CL, cardiac lobe; GS, genal spine; IR, interophthalmic region; MTL, medial thoracetronic and pleural lobes (poorly discernible); OR, ophthalmic ridge; PPL, posterior prosomal lobe; TF, thoracetronic flange; TTP, terminal thoracetronic projection forming ‘swallowtail’. Lined polygons and the thick dashed line (running over the thoracetron and proximal part of telson) in (B) represent artefacts of latex casting, with dark-grey lines for convex artifacts and light-grey lines for concave artifacts. Scale bars = 10 mm. Image converted to greyscale. Photo credit: Jonatan Audycki.

**Figure 6:**
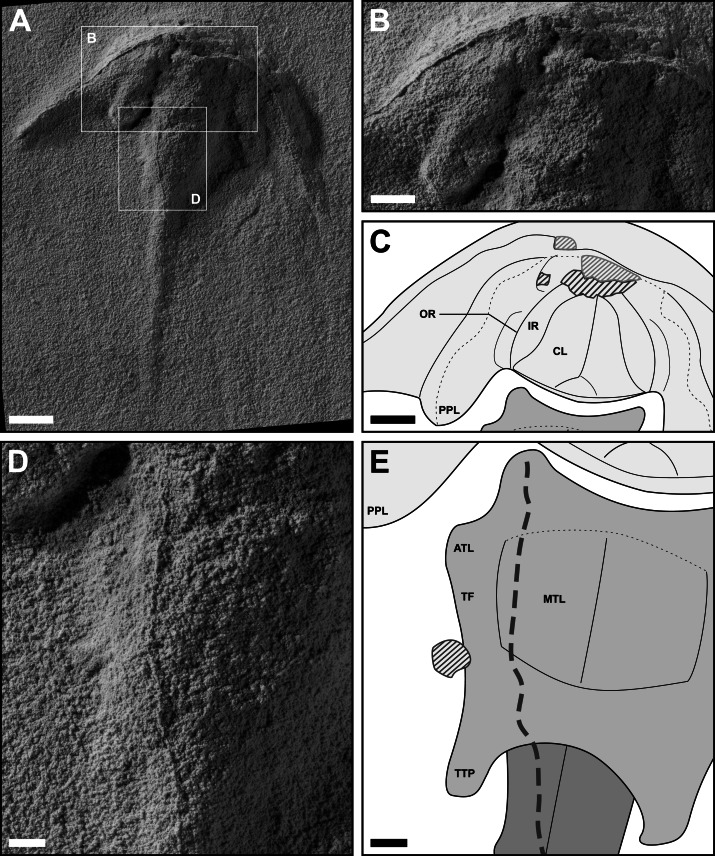
Dorsal prosomal and thoracetronic features of *Polonolimulus zaleziankensis* gen. et sp. nov. (latex peel gz4142 of holotype Muz. PGI 1808.II.10). (A) Latex peel gz4142 oriented and lit so as to highlight the relevant morphological features, with white rectangles showing the enlarged areas in (B) and (D). Image lit with low-angle light from top-left. (B) Close-up of the central part of prosoma. (C) Interpretative drawing of (B). (D) Close-up of the left part of thoracetron. (E) Interpretative drawing of (D). Acronyms: ATL, anterolateral thoracetronic lobe; CL, cardiac lobe; IR, interophthalmic region; MTL, medial thoracetronic and pleural lobes (poorly discernible); OR, ophthalmic ridge; PPL, posterior prosomal lobe; TF, thoracetronic flange; TTP, terminal thoracetronic projection forming ‘swallowtail’. Lined polygons in (C) and (E), and the thick dashed line in (E) represent artifacts of latex casting, with dark-grey lines for convex artifacts and light-grey lines for concave artifacts. Scale bars: (A): 10 mm; (B, C): 5 mm; (D, E): 2 mm. Images converted to greyscale. Photo credit: Jonatan Audycki.

**Holotype:** Muz. PGI 1808.II.10 (Figs. 3, 5, 6, S4; additional images available at OSF: https://doi.org/10.17605/OSF.IO/632RU).

**Figure 7:**
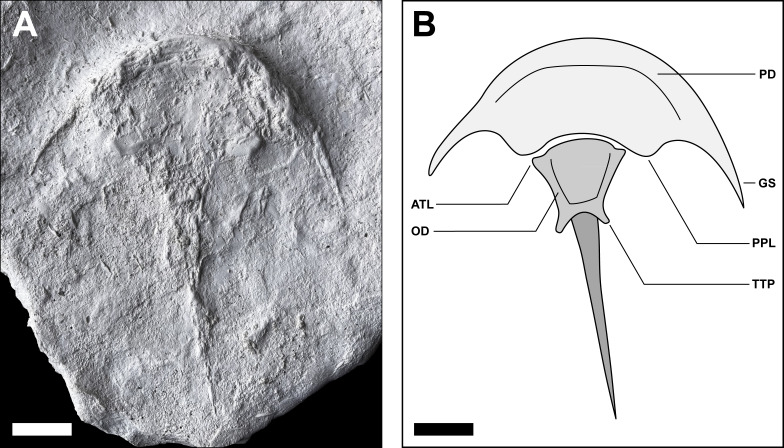
Paratype of *Polonolimulus zaleziankensis* gen. et sp. nov. Muz. PGI 1808.II.11 visible in ventral view. (A) Gypsum cast of the original concave fossil; light from top left. (B) Interpretative drawing of Muz. PGI 1808.II.11 highlighting main features; arrow shows the position of genal spine kink on the left side of the specimen. Acronyms: ATL, anterolateral thoracetronic lobe; GS, genal spine; OD, opisthosomal doublure; PD, prosomal doublure; PPL, posterior prosomal lobe; TTP, terminal thoracetronic projection forming ‘swallowtail’. Scale bars: 10 mm. Image converted to greyscale. Photo credit: Grzegorz Niedźwiedzki.

**Paratype**: Muz. PGI 1808.II.11 (Fig. 7).

**Type locality and horizon:** Zalezianka-Gózd outcrop near Zalezianka, Holy Cross Mountains, southern Poland (50°58′56″N, 20°45′06″E); Zagnańsk/Goleniawy Formation, Lower/Middle Buntsandstein, uppermost Induan/lowermost Olenekian, Lower Triassic (see ‘Geological setting’ for correlation details).

**Material:** Two articulated whole-body specimens preserved as concave impressions in sandstone. Holotype Muz. PGI 1808.II.10 preserved on the (inferred) upper surface of a rock-slab; another, convex cast visible on the lower surface of the slab, likely representing a trace fossil (Fig. 3). Paratype Muz. PGI 1808.II.11 only available as a gypsum cast of the original fossil (Fig. 7).

**Diagnosis:** Austrolimulid with kinked, elongated, postero-laterally oriented genal spines extending almost to the posterior margin of thoracetron; posterior prosomal margin extending into a pair of posteriorly directed projections, located laterally of the lateral thoracetronic margins; prosoma slightly longer along midline than thoracetron.

**Description:** Holotype Muz. PGI 1808.II.10 (based on latex peel gz4142): An articulated and mostly complete specimen in dorsal view (Fig. 5). Specimen approximately 84 mm long as preserved and 71 mm wide between the genal spine tips; left side seemingly slightly better preserved than right side.

Prosoma crescentic in outline, 19 mm long at midline (PL_1_) and 34 mm long at level of genal spine tips (PL_2_). Genal spines kinked abaxially, with kink located approximately 3/5 of distance from anterior midpoint of prosoma to genal spine tip; genal spines narrowing gradually from that point until slightly widening distally (Figs. 5, 6A). Genal spine kink and narrowing of genal spine much more distinct on left side than on right. Genal splay (α) ca. 47.5 degrees for left genal spine and 42 degrees for right. Prosomal width from organismal midline to genal spine tip (PW_1_) 36 mm on left side and 32 mm on right side. Posterior prosomal margin anteriorly arching from genal spine tips adaxially beyond genal spine kink, then sharply extending posteriorly, producing a pair of posteriorly-directed projections—posterior prosomal lobes—located laterally of thoracetronic margins. Distance from posterior prosomal lobe terminus to organismal midline (PW_2_) approximately 14 mm on left side and 17 mm on right. The posterior prosomal margin extending anteriorly from the distal tips of posterior prosomal lobes and arching adaxially and slightly posteriorly at the level of genal spine kink. Prosoma unevenly vaulted adaxially from lateral margins of posteriorly-directed projections; convexity of prosoma lateral regions less pronounced. Ophthalmic ridges, if inferred correctly, moderately arching anteriorly and adaxially, with anterior structure obscured by poor preservation. Cardiac lobe poorly discernible, subtriangular in shape, with hardly distinct median crest possibly present (Fig. 6B).

Thoracetron trapezoidal, ca. 14 mm long at midline (TL_1_). Lateral thoracetronic margins arching abaxially in the anterior part, creating a small, rounded anterolateral thoracetronic lobes on each side, then arching adaxially in posterior thoracetron part (Figs. 5, 6D). Thoracetron width from midline to anterolateral thoracetronic lobe tips (TW_1_) 8.5 mm on left side, 10 mm on right side. Terminal thoracetron expressed as blunt projections forming a small ‘swallowtail’, extending thoracetron length from anterior thoracetronic margin (TL_2_) to 16.5 mm. Thoracetron width from midline to maximum lateral extent of posterior thoracetronic projections (TW_2_) 6 mm on left side, 7 mm on right side. Thoracetronic flange present (Figs. 6A, 6D). Medial thoracetronic lobe moderately vaulted, but poorly discernible from pleural lobes; axial furrows and apodemes not preserved. Presence of medial thoracetronic ridge uncertain. Distinct flattened region present in posterior thoracetron part between medial lobe and terminal ‘swallowtail’ (Figs. 5, S4). Fixed or movable spines and notches not observed.

Telson with pronounced keel, approximately 45 mm long (TeL) as preserved; preservation gradually poorer in distal half, distal tip of telson possibly missing (Figs. 5, 6A).

Paratype Muz. PGI 1808.II.11 (gypsum cast of the original specimen): An articulated complete specimen seemingly in ventral view. Specimen approximately 128 mm long as preserved and 103 mm wide between the genal spine tips. Body margins preserved three-dimensionally, medial areas flat (Fig. 7).

Prosoma crescentic, 34 mm long at midline (PL_1_) and ca. 49 mm long at level of genal spine tips (PL_2_). Abaxial genal spine kink not visible on right side of specimen, but discernible on left side and similarly to holotype located at ∼3/5 of distance from anterior midpoint of prosoma to genal spine tip. Genal splay (α) ca. 47 degrees for left genal spine and 43 degrees for right. Prosomal width from midline to genal spine tip (PW_1_) 52 mm on both sides. Posterior prosomal margin extending into posterior prosomal lobes; prosomal width from midline to posterior prosomal lobe terminus (PW_2_) 22 mm on left side and 20 mm on right side. Medial part of three-dimensionally preserved anterior prosomal margin extending posteriorly into a wide fringe, possibly prosomal doublure.

Thoracetron trapezoidal, 25 mm long at midline (TL_1_) and 30 mm to terminal thoracetronic projection terminus (TL_2_). Anterolateral thoracetronic lobes poorly discernible; if inferred correctly, thoracetron width from midline to anterolateral thoracetronic lobe tips (TW_1_) 17 mm on left side, 12 mm on right side. Thoracetron width from midline to maximum lateral extent of posterior thoracetronic projections (TW_2_) 9 mm on left side, 6 mm on right side. Posterior and lateral thoracetronic margins preserved three-dimensionally as thick rims, possibly opisthosomal doublure.

Telson seemingly complete, 68 mm long (TeL) as preserved (Fig. 7).

**Remarks:** *Polonolimulus zaleziankensis* is the first horseshoe crab described from the Polish Triassic and the third xiphosurid occurrence from Poland overall, after *Limulitella* cf. *liasokeuperinus* (Braun, 1860) from the Lower Jurassic sandstone near Szydłowiec, north of Skar.zysko-Kamienna and *Crenatolimulus darwini* (Kin & Błazejowski, 2014) from the Upper Jurassic Owadów-Brzezinki Konservat-Lagerstätte (Karaszewski, 1975; Kin & Błazejowski, 2014; Bicknell et al., 2021a). As the hypertrophied genal spines of *P. zaleziankensis* are comparable to other horseshoe crabs assigned to Austrolimulidae, consideration must be given to known austrolimulids, especially those of similar age and occurring from within or nearby the Central European Basin System. The closest temporally and geographically is *Psammolimulus gottingensis* (Lange, 1922) from the Lower Triassic of Germany (Fig. S5A; Bicknell & Shcherbakov, 2021). The thoracetron of *Polonolimulus* is comparable to *Psammolimulus* in shape and relative size, although *Polonolimulus* lacks the enlarged posterior pleural spines. The prosomal morphologies differ considerably, with the genal spines oriented posteriorly in *Psammolimulus,* while extending much more laterally in *Polonolimulus*. The same applies when comparing *Polonolimulus* with *Attenborolimulus superspinosus* Bicknell & Shcherbakov, 2021 from the Lower Triassic of Russia (Fig. S5B). The only other austrolimulids from Europe, excluding the occurrences assigned to genus *Limulitella* Størmer, 1952 of contentious systematic position, are *Batracholimulus fuchsbergensis* (Hauschke & Wilde, 1987) and *Franconiolimulus pochankei* Bicknell, Hecker & Heyng, 2021 from the Upper Triassic and Lower Jurassic of Germany (Figs. S5C, S5D). In both cases, the genal spines are much shorter than in *Polonolimulus*.

When comparing the new material to other Triassic xiphosurids known from Europe and vicinity, consideration must also be given to the genus *Limulitella*, which was retrieved as an austrolimulid in the phylogenetic analysis of Lamsdell (2020) and therefore potentially a close relative of *Polonolimulus*. However, Bicknell & Shcherbakov (2021) and Bicknell et al. (2021a) argued against this hypothesis, pointing out morphological similarities between *Limulitella* and many limulids, as well as overall closer position of this genus in morphospace to representatives of Limulidae. The morphology of *Limulitella bronni* Schimper, 1853 (Fig. S5E), *Limulitella volgensis* Ponomarenko, 1985 (Fig. S5F) and *Limulitella tejraensis* Błażejowski et al., 2017 (Fig. S5G) is rather typical for limulids, lacking either the hypertrophied genal spines or the reduced size of thoracetron typical for members of Austrolimulidae. However, in *Limulitella liasokeuperinus* (Braun, 1860) genal spines are more elongated and thus closer to the austrolimulid condition. As such, a detailed comparison of *Polonolimulus* with *Limulitella* cf. *liasokeuperinus* from the Lower Jurassic of Holy Cross Mountains would be relevant. Unfortunately, the only specimen was lost in the bombing of Warsaw in September 1939 and no illustration or description of it is known, other than “it was the size of a hand and quite well preserved” (Karaszewski, 1975; in Polish). As the *Limulitella liasokeuperinus* holotype has also been lost, only a single cephalothorax of *Limulitella* cf. *liasokeuperinus* is available for comparison (Fig. S5H; Hauschke & Wilde, 1984). Its narrow prosomal outline and posteriorly oriented genal spines without any lateral direction clearly separate this specimen from *Polonolimulus*.

Outside of Europe, austrolimulids have been reported from North America and Australia (Bicknell & Pates, 2020). *Vaderlimulus tricki* Lerner, Lucas & Lockley, 2017 from the Lower Triassic Thynes Group of Idaho, USA is of comparable age and quite similar morphology to *Polonolimulus*, especially in the genal splay and size proportion of prosoma to thoracetron (Lerner & Lucas, 2022). However, the two taxa differ considerably in prosomal shape, with genal spines of *Polonolimulus* exhibiting a slender, slightly kinked morphology distinctly produced from the medial part of the prosoma, while in *Vaderlimulus* genal spines are broad, direct extensions of the prosoma without genal kinks (Fig. S5I). Stratigraphically close to *Polonolimulus* is *Tasmaniolimulus patersoni* (Bicknell, 2019) from the Lower Triassic Jackey Shale of Tasmania, Australia (Bicknell et al., 2022b). However, its morphology differs significantly, with the genal spines of *Tasmaniolimulus* directed fully posteriorly, without the genal splay of *Polonolimulus* (Fig. S5J). *Polonolimulus* also differs clearly from *Dubbolimulus peetae* Pickett, 1984 from the Middle Triassic Napperby Formation of Australia, with the latter lacking any elongation of genal spines (Fig. S5K). The prosomal morphology of *Austrolimulus fletcheri* Riek, 1955 from the Middle Triassic Hawkesbury Sandstone of Australia (Fig. S5L) is very comparable to that of *Polonolimulus*, with both taxa exhibiting elongated, slender genal spines with large splay. The thoracetron morphology is also broadly similar, although the poor preservation of *Polonolimulus* material excludes the identification of caudal segments present in *Austrolimulus*. At the same time, *Polonolimulus zaleziankensis* differs from *A. fletcheri* in a smaller genal spine splay and a larger thoracetron in *P. zaleziankensis*. Additionally, the *P. zaleziankensis* posterior prosomal margin has two rounded posteriorly directed extensions—posterior prosomal lobes—that are distinct from possibly homologous structures in *A. fletcheri*. For these reasons, we consider the *Polonolimulus zaleziankensis* material sufficiently distinct to merit its separation into a new genus, rather than a new species of *Austrolimulus*.

The most distinct feature of *Polonolimulus* is the prominent, posteriorly-directed extension of posterior prosomal margin—the posterior prosomal lobes. Similar structures, comparable in size relative to the body, have been reported from Carboniferous belinurids and reflect extensions of ophthalmic ridges into ophthalmic spines (Lamsdell, 2020). The posterior prosomal extensions in *Polonolimulus* could therefore be interpreted as ophthalmic spine protrusions, convergent with belinurids. However, belinurid ophthalmic spines are more adaxially positioned and extend over the thoracetron, which is distinct from *Polonolimulus* prosomal lobes that are located abaxially and lateral to thoracetronic margins. We therefore interpret the posterior prosomal lobes as distinct from ophthalmic ridges and most likely homologous with the posteriorly-directed processes present in *Austrolimulus* (Riek, 1955; Riek, 1968). The three-dimensional, ridge-like extensions in Muz. PGI 1808.II.10 are therefore interpreted as a taphonomic structures, likely reflecting rapid decay and subsequent partial collapse of the cardiac lobe.

On the underside of the slab preserving the *Polonolimulus zaleziankensis* holotype is a convex xiphosurid-shaped impression (Figs. 3B, 3D). The holotype Muz. PGI 1808.II.10 is preserved as a concave impression ∼10 mm deep, while the slab is ≥50 mm thick, precluding the possibility that the convex structure is the same fossil. However, the considerable similarities between the two specimens in size and overall shape (Muz. PGI 1808.II.10: total length–84 mm, max prosomal width–71 mm, max thoracetron width–18.5 mm; convex impression on the lower surface of the slab: total length–ca. 84 mm, max prosomal width–ca. 70 mm, max thoracetron width–ca. 17 mm) and their positions within the rock slab (one almost perfectly below and only slightly rotated in regard to the other) make it unlikely that they are completely unrelated. It is possible that the convex structure represents an escape trace fossil produced by the individual that is preserved as a body impression on the slab’s upper surface, *i.e.,* a taphichnia *sensu* Pemberton, MacEachern & Frey (1992)—an unsuccessful escape attempt. The poorer preservation of the trace fossil would be consistent with the animal’s movements and sediment shifting during its escape attempt, as the more detailed concave impression reflects more gradual infilling of the space left by the decaying body at the sediment interface. For the carcass to be preserved as a concave impression in dorsal view on the upper surface of the slab, the body had to lay on its dorsal side, suggesting the animal was overturned during or immediately prior to its escape attempt. This interpretation is highly speculative and requires further examination, potentially using non-invasive imaging technology, such as computed tomography, synchrotron or neutron imaging. Therefore, the convex structure from the lower surface of the rock slab is currently excluded from the description of *P. zaleziankensis*.

**Occurrence:** Only known from the type locality.

### Geometric morphometrics of fossil xiphosurids from the Triassic

**Figure 8:**
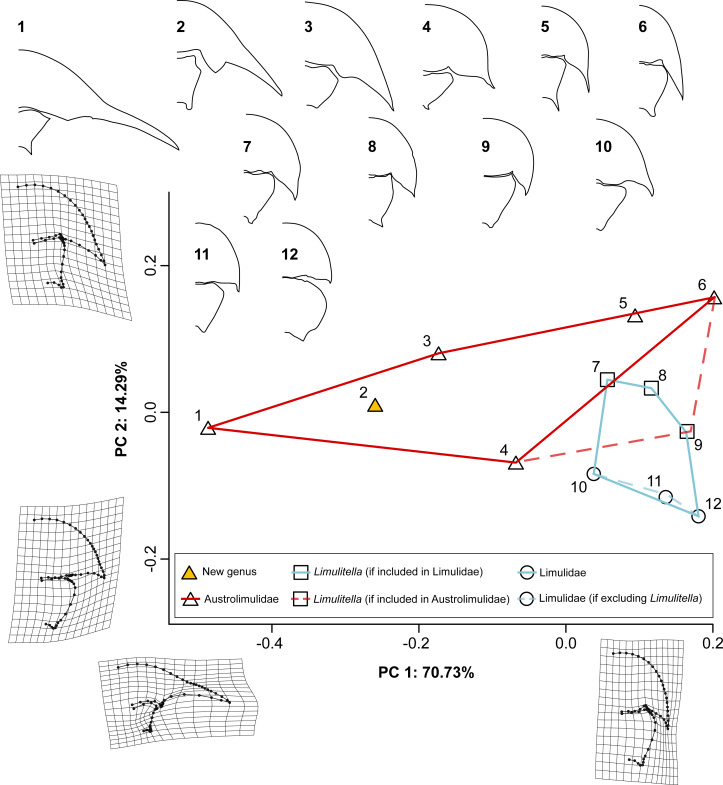
PCA plot of Triassic xiphosurids using both prosomal and thoracetronic data. TPS grids show deformation between the average and the minimum and maximum landmark coordinates for PC1 and PC2. Convex hulls for Austrolimulidae (red) and Limulidae (light blue) are plotted in two alternative ways: solid lines for the inclusion of *Limulitella* is in Limulidae, and dashed lines if *Limulitella* is included within Austrolimulidae. Outlines of the specimens used in the analysis are shown above the plot: 1–AM F38274 *Austrolimulus fletcheri*, 2–gz4142 (latex peel of the holotype Muz. PGI 1808.II.10) *Polonolimulus zaleziankensis*, 3–UCM 140.25 *Vaderlimulus tricki*, 4–MMF 27693 *Dubbolimulus peetae*, 5–GZG.INV.45730a *Psammolimulus gottingensis*, 6–PIN 5640/220 *Attenborolimulus superspinosus*, 7–UNISTRA.2015.0.50968 *Limulitella bronni*, 8–LIM 68 *L. bronni*, 9–ZPAL V.46/101 *Limulitella tejraensis*, 10–PMSL T-993 *Sloveniolimulus rudkini*, 11–MGSB M 262 *Tarracolimulus rieki*, 12–MGSB 19195 *Heterolimulus gadeai*. Outlines are not to scale.

The PCA of Triassic xiphosurids using prosomal and thoracetronic data reveals that 85.02% of shape variation is explained by the first two principal components (Fig. 8). PC1 (70.73% shape variation) describes both the abaxial position of the genal spine tip and the thoracetron size relative to prosoma. Taxa bearing strongly splayed genal spines and relatively small thoracetrons are located on the far end of the negative PC1 space (−0.487 for *Austrolimulus fletcheri*, −0.260 for *Polonolimulus zaleziankensis,* −0.173 for *Vaderlimulus tricki*), while taxa with short or predominantly posteriorly directed genal spines and large thoracetrons occupy the more positive PC1 space (0.202 for *Attenborolimulus superspinosus*, 0.180 for *Heterolimulus gadeai*, 0.165 for *Limulitella tejraensis*). PC2 explains a much smaller percentage of shape variation (14.29%) and likely reflects the thoracetron shape, particularly the structure and position of anterior thoracetronic margin.

**Figure 9:**
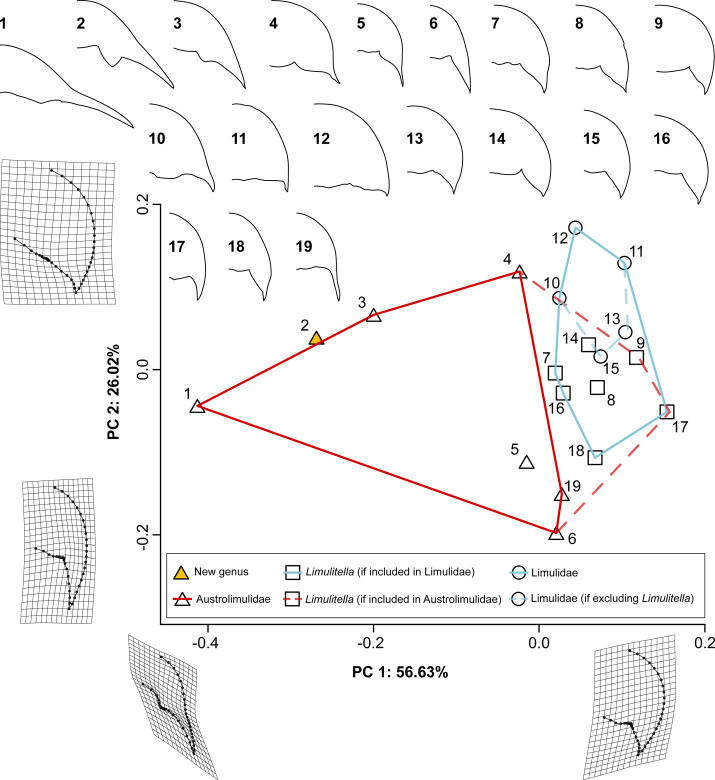
PCA plot of the Triassic horseshoe crabs using prosomal data only. TPS grids show deformation between the average and the minimum and maximum landmark coordinates for PC1 and PC2. Convex hulls for Austrolimulidae (red) and Limulidae (light blue) are plotted in two alternative ways: solid lines for the inclusion of *Limulitella* is in Limulidae, and dashed lines if *Limulitella* is included within Austrolimulidae. Outlines of the specimens used in the analysis are shown above the plot: 1–AM F38274 *Austrolimulus fletcheri*, 2–gz4142 (latex peel of the holotype Muz. PGI 1808.II.10) *Polonolimulus zaleziankensis*, 3–UCM 140.25 *Vaderlimulus tricki*, 4–MMF 27693 *Dubbolimulus peetae*, 5–GZG.INV.45730a *Psammolimulus gottingensis*, 6–PIN 5640/220 *Attenborolimulus superspinosus*, 7–UNISTRA.2015.0.50968 *Limulitella bronni*, 8–LIM 68 *L. bronni*, 9–ZPAL V.46/101 *Limulitella tejraensis*, 10–PMSL T-993 *Sloveniolimulus rudkini*, 11–MGSB M 262 *Tarracolimulus rieki*, 12–MGSB 19195 *Heterolimulus gadeai*.: 13–MAN 8240 *Keuperlimulus vicensis*, 14–ZPAL V.46/120 *Limulitella tejraensis*, 15–MB.A.0207 Limulidae indet. (‘*Limulus kieri*’), 16–ZPAL V.46/106 *L. tejraensis*, 17–SNSB-BSPG 1967 XVI 27 *Limulitella* cf. *liasokeuperinus*, 18–ZPAL V.46/103p *L. tejraensis* , 19–UTGD 123979 *Tasmaniolimulus patersoni*. Outlines are not to scale.

In the PCA variant using prosomal data only, the two first principal components explain 82.65% of shape variation (Fig. 9). Similarly to the first variant, the PC1 (56.63% shape variation) describes the abaxial position of the genal spine tip, with taxa characterized by the largest splay placed on the negative edge of PC1 space (−0.413 for *Austrolimulus fletcheri*, −0.269 for *Polonolimulus zaleziankensis,* −0.200 for *Vaderlimulus tricki*), and taxa with minimal lateral expansion of genal spines positioned in positive PC1 space (0.155 for *Limulitella liasokeuperinus*, 0.118 for *L. tejraensis*, 0.104 for *Keuperlimulus vicensis*, 0.103 for *Tarracolimulus rieki*). PC2 (26.02% shape variation) predominantly details how the genal spine extends the prosomal outline posteriorly: specimens with elongated, posteriorly directed genal spines are located in negative PC2 space (−0.198 for *A. superspinosus*, −0.151 for *Tasmaniolimulus patersoni*, −0.112 for *Psammolimulus gottingensis*, −0.107 for specimen ZPAL V.46/103p *L. tejraensis*), while taxa with reduced genal spines are located in positive PC2 space (0.17176713 for *Heterolimulus gadeai*, 0.129 for *Tarracolimulus rieki,* 0.119 for *Dubbolimulus peetae*).

Austrolimulids occupy a much larger portion of morphospace than limulids in both analyses, regardless of the inclusion of *Limulitella*. Limulids are always located in positive PC1 space, but vary along PC2. Specimens assigned to *Limulitella* are distributed around neutral PC2 space in both analyses. The exclusion of *Limulitella* from Limulidae results in the family occupying strictly negative PC2 space in the prosomal and thoracetronic data variant, and neutral-positive PC2 space in the prosoma-only data variant of the analysis.

In both analyses, *Polonolimulus zaleziankensis* is located in very negative PC1 space

(−0.260 for prosoma+thoracetron variant, −0.269 for prosoma-only variant) and in neutral PC2 space (0.010 for prosoma+thoracetron, 0.039 for prosoma only variant), thus falling proximal to or within austrolimulid morphospace. In both cases it is located between *Austrolimulus fletcheri* and *Vaderlimulus tricki*.

### Palaeobiogeography of Triassic horseshoe crabs

**Figure 10:**
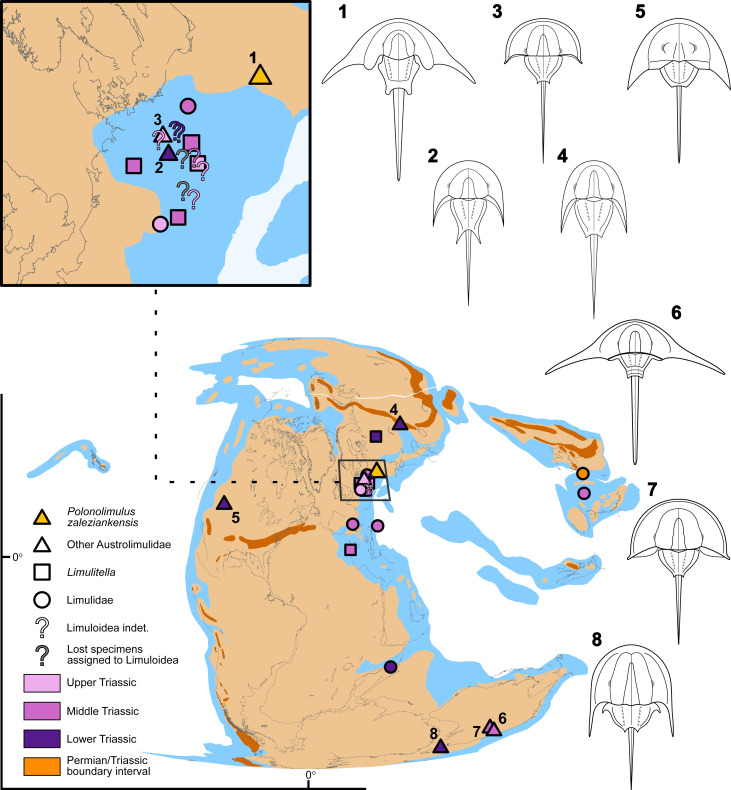
Palaeobiogeography of Triassic horseshoe crabs. Palaeogeography of the world is reconstructed at 245 Ma (Anisian, Middle Triassic) and displayed using Mollweide projection, with a close-up of the Central European Basin System. The extents of shallow seas (light blue), landmasses (pale yellow) and mountain ranges (orange) are reconstructed based on Cao et al. (2017). Symbols represent Triassic localities that have yielded horseshoe crab fossils, their ages, and taxonomic assignment. The palaecoordinates of the xiphosurid localities are also reconstructed at 245 Ma for consistency. Simplified reconstructions of austrolimulids are displayed for context and comparison: 1–*Polonolimulus zaleziankensis* from the Lower Triassic (uppermost Induan/lowermost Olenekian) Zalezianka-Gózd locality, Poland; 2–*Psammolimulus gottingensis* from the Lower Triassic (Olenekian) Solling Formation, Germany; 3–*Batracholimulus fuchsbergensis* from the Upper Triassic (Norian/Rhaetian boundary) Exter Formation, Germany; 4–*Attenborolimulus superspinosus* from the Lower Triassic (upper Olenekian) Petropavlovka Formation, Russia; 5–*Vaderlimulus tricki* from the Lower Triassic (lower Spathian, Olenekian) Thaynes Group, USA; 6–*Austrolimulus fletcheri* from the Middle Triassic (lower Anisian) Beacon Hill Quarry, Hawkesbury Sandstone, Australia; 7–*Dubbolimulus peetae* from the Middle Triassic (lower Anisian) Ballimore Formation, Australia; 8–*Tasmaniolimulus patersoni* from the Lower Triassic (lower Induan) Jackey Shale, Tasmania (Australia). Since the investigated xiphosurid localities differ stratigraphically, their geographical position relative to continental boundaries and shallow seashores are approximated. The display window on the map showing close-up boundaries is not square due to employed map projection. For the full list of investigated localities along with their current and reconstructed palaeocoordinates and temporal data sources see Table S2.

Palaeobiogeographic reconstruction reveals that during the Triassic horseshoe crabs were widely distributed in the eastern parts of Pangea (predominantly along the coast) and further east in South China Block while only a single taxon, *Vaderlimulus tricki,* was reported from western Pangea (Fig. 10). Austrolimulid occurrences designate the northern, western and southern limits of the Triassic xiphosurid geographic range, while the eastern limits are designated by limulid occurrences. Palaeocoordinates of the Zalezianka-Gózd locality were reconstructed as 28.3234°N, 31.0043°E, which places *Polonolimulus zaleziankensis* in the northern part of the Triassic xiphosurid range.

## Discussion

**Figure 11:**
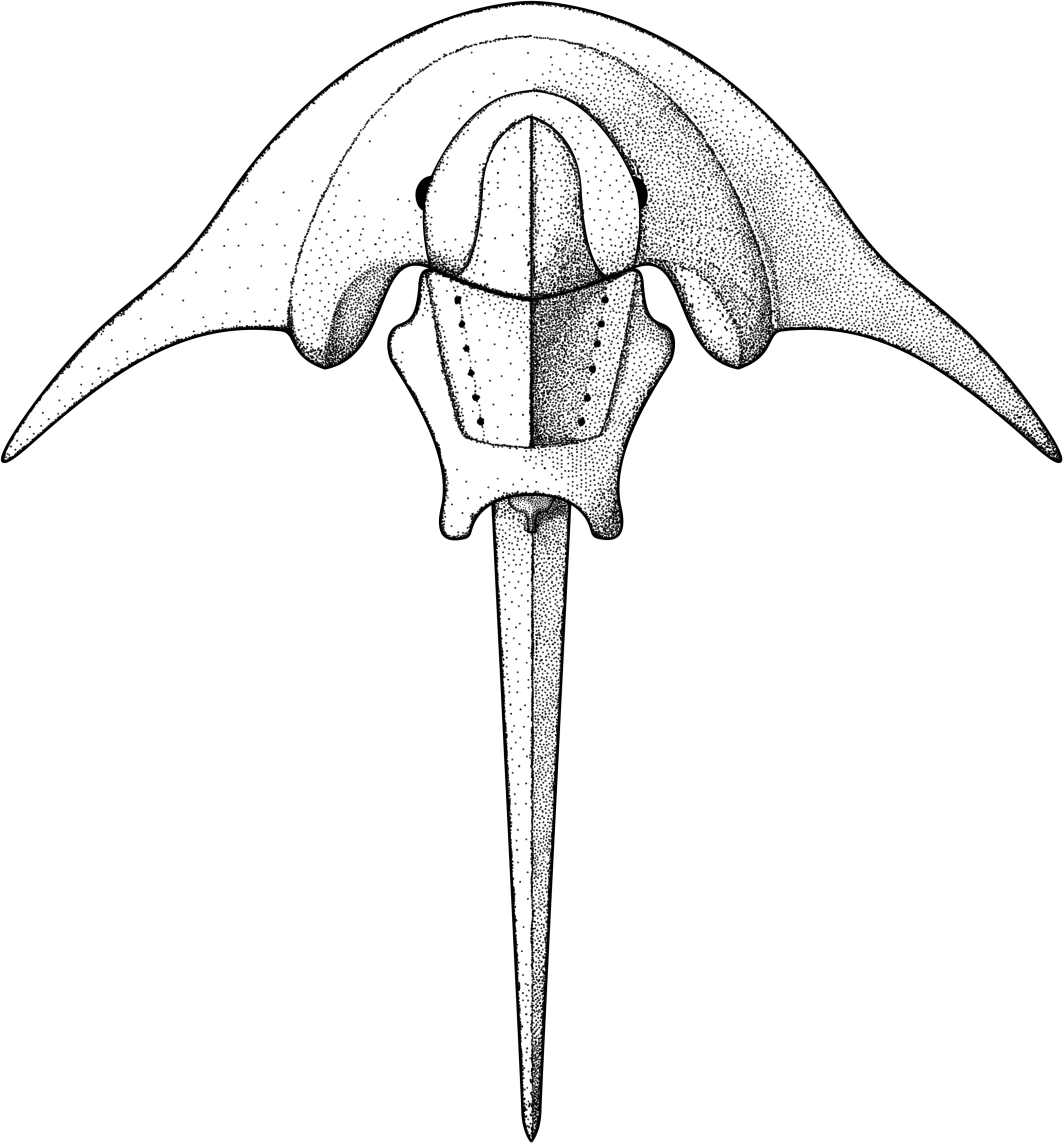
Idealized reconstruction of *Polonolimulus zaleziankensis* gen. et sp. nov. Reconstruction credit: Jonatan Audycki.

The new xiphosurid material from the Early Triassic of Poland displays one of the most ‘extreme’ austrolimulid morphologies in terms of genal spine elongation and splay (Fig. 11), thus significantly extending the geographical range of such forms previously known only from Australia and North America (Bicknell & Pates, 2020). Moreover, while it is uncertain whether the Zalezianka-Gózd locality was lagoonal or deltaic, the marine influence is evident (*e.g.*, the presence of marine bivalves), contrasting with most other austrolimulids reported from brackish or even fully freshwater settings (Lamsdell, 2016; Bicknell & Shcherbakov, 2021). It is therefore particularly interesting that *Polonolimulus zaleziankensis* bears great similarity to *Austrolimulus fletcheri* from the reportedly freshwater Middle Triassic Beacon Hill locality in Australia, made even more surprising given the great geographical distance between the reconstructed palaeocoordinates of their occurrences (over 13,500 km). If the similarity in general morphology suggests their close relationship, such extensive distribution could be reasonably accepted for marine taxa, but would be more difficult to explain for strictly freshwater animals. The occurrences of both *Austrolimulus* and *Polonolimulus* close to the reconstructed shorelines also support their connection to fully marine habitats (Fig. 10). Furthermore, the morphologically similar austrolimulid *Vaderlimulus tricki* is also known from a marine setting (Lerner, Lucas & Lockley, 2017; Lerner & Lucas, 2022). Indeed, the assumption that colonization and adaptation to freshwater environments by austrolimulids is required to explain the occurrences of their fossils in non-marine settings seems potentially unnecessary in case of *Austrolimulus*, when the modern horseshoe crabs are known to venture up rivers on occasion, with *Carcinoscorpius rotundicauda* (Latreille, 1802) in particular being observed as far as 150 km inland (Annandale, 1922). While this is an exceptional case facilitated by the specific character of the brackish, tidally influenced Hoogly River (Chugh, 1961), it shows the potential for finding marine/brackish xiphosurids in apparently non-marine deposits. Since most examined occurrences of Triassic xiphosurids are also reconstructed either within or proximal to the shallow marine areas, it is prudent to reinvestigate whether the available data supports their alleged fully-freshwater lifestyle. Such an examination might highlight that some brackish/marine forms may have occasionally ventured further inland.

The broad geographic range of the first austrolimulids, widely distributed along different shores of Pangea in the Early Triassic, is impressive (Fig. 10). This could be interpreted either as a rapid diversification and dispersal immediately following the end-Permian extinction, or as an indication that austrolimulids originated in the Late Permian, or possibly even earlier. Phylogenetic analysis of Lamsdell (2020) retrieved some Palaeozoic xiphosurids as basal austrolimulids: the Permian *Shpineviolimulus jakovlevi* Bicknell, Naugolnykh & Birch, 2020 and *Panduralimulus babcocki* Allen & Feldmann, 2005, and Carboniferous *Boeotiaspis longispinus* (Schram, 1979). However, none of them display the hypertrophied genal spines and reduced thoracetron size characteristic of the Austrolimulidae, precluding an unequivocal assignment of any of these species as austrolimulids or their close relatives. Nonetheless, a Palaeozoic origin of austrolimulids and their subsequent survival through the end-Permian extinction cannot be ruled out, particularly since recent discoveries of complex ecosystems thriving just after end-Permian extinction demonstrate that earlier hypotheses regarding its extent and the hostile conditions in its aftermath might have been exaggerated (Dai et al., 2023; Peng et al., 2025). Currently, both hypotheses on the timing of origin and early dispersal of austrolimulids remain speculative and require further study. Regardless of whether austrolimulids first appeared in Palaeozoic or Early Triassic, extinction of many other marine animals and resulting vacant ecological space certainly contributed to the quick diversification and dispersal of the morphologically ‘extreme’ xiphosurids (Bicknell & Shcherbakov, 2021).

The wide distribution of Early Triassic austrolimulids makes it difficult to clearly determine which among them represents the morphology closest to ancestral. The oldest species is *Tasmaniolimulus patersoni* from the Induan Jackey Shale in Tasmania (Bicknell et al., 2022b). *Tasmaniolimulus* was also retrieved as the most basal austrolimulid in the phylogenetic analysis of Lamsdell (2020). The same analysis retrieved *Limulitella* as sister taxon to *Psammolimulus*, together forming clade more derived than *Tasmaniolimulus* within Austrolimulidae. However, others argued that *Limulitella* lacks major austrolimulid features and should be considered either a member of Limulidae, or perhaps a transitional form from between limulids and austrolimulids (Bicknell & Shcherbakov, 2021; Bicknell et al., 2021a). The latter view is somewhat supported by the results of PCA performed in this study, since specimens assigned to *Limulitella* fall into morphospace intermediate between Austrolimulidae and Limulidae (Figs. 8 and 9). However, neither the position in morphospace nor similarities in general shape are readily informative of phylogenetic relationships, which should be further investigated with phylogenetic methods. There is also considerable variation within *Limulitella* itself, likely due to ontogeny, as evidenced by the largest examined specimen of *Limulitella tejraensis* being closer on the PCA plot to *Tasmaniolimulus* than other *L. tejraensis* specimens (Fig. 9). More data is needed on the ontogeny of Triassic xiphosurids. The exact taxonomic composition of *Limulitella* is also uncertain, necessitating further studies focusing on this genus (Lamsdell & Ocon, 2025).

Austrolimulids are not uniformly distributed in morphospace. The most morphologically ‘extreme’ are Early and Middle Triassic forms with elongated and splayed genal spines: *Vaderlimulus, Austrolimulus* and the new *Polonolimulus*, which , fall within negative PC1 space (Figs. 8 and 9). The second austrolimulid subgroup consists of the exclusively Early Triassic taxa with elongated genal spines that lack a notable splay: *Psammolimulus*, *Attenborolimulus* and *Tasmaniolimulus*, and is located in more positive PC1 space (Fig. 9). The last examined austrolimulid, the Middle Triassic *Dubbolimulus peetae* falls between the two earlier subgroups in the PC1 space. *Dubbolimulus peetae* falls into very positive PC2 space, separate from other species (Figs. 8 and 9). If limulid-like morphology is assumed to be more basal (*e.g.*, based on the proposed Carboniferous limulid *Albalimulus bottoni* (Bicknell & Pates, 2019), the Permian/Triassic ?limulid *Guangyuanolimulus shangsiensis* (Hu et al., 2022), and the similarities between limulids and paleolimulids), the morphology of *Tasmaniolimulus* subgroup might be considered more derived than that of the limulid-like *Limulitella*, and is in turn less derived than both the most ‘extreme’ austrolimulids and *Dubbolimulus*. The lack of axial thoracetronic segmentation in *Austrolimulus* and *Vaderlimulus* also supports their morphology as the more derived within Austrolimulidae (Lamsdell & Ocon, 2025). The lack of axial thoracetronic segmentation in *Limulitella* could therefore be interpreted as an independent loss. Because of poor preservation of specimens it is uncertain whether several other Triassic xiphosurids, including *Psammolimulus*, *Dubbolimulus* and *Batracholimulus* displayed axial thoracetronic segmentation (Lamsdell & Ocon, 2025). Detailed re-examination of all available material and the combination of new phylogenetic, ontogenetic and taphonomic data will be necessary for further studies on austrolimulid evolution.

## Conclusions

We present here *Polonolimulus zaleziankensis* gen. et sp. nov., a new austrolimulid horseshoe crab from the Lower Triassic brackish or marginal-marine deposits of Holy Cross Mountains, Poland. The PCA results demonstrate that austrolimulids are grouped into three morphologically distinct clusters, with the new taxon falling into the morphologically most ‘extreme’ group, characterized by greatly elongated and splayed genal spines. Furthermore, *Limulitella* is recovered as morphologically intermediate between Limulidae and Austrolimulidae. Our palaeobiogeographic reconstruction shows an almost global distribution of austrolimulids already in the Early Triassic, which could suggest an earlier dispersal in Late Permian and/or a rapid diversification of the group in the earliest Triassic. The occurrences of *Polonolimulus* and the morphologically most similar *Austrolimulus* are within or proximal to shallow-marine areas, suggesting dispersal through a fully marine environment. The unique morphology of *P. zaleziankensis* adds to the disparity of known Triassic xiphosurids, further demonstrating a considerable diversity of this group in the early Mesozoic.

## Supplemental Information

10.7717/peerj.20950/supp-1 Supplemental Information 1 Specimens included in the geometric morphometric analysis

10.7717/peerj.20950/supp-2 Supplemental Information 2 Localities included in the palaeobiogeographic reconstruction

10.7717/peerj.20950/supp-3 Supplemental Information 3 Code and shapefiles for the palaeobiogeographic reconstruction of Triassic xiphosurid occurences

10.7717/peerj.20950/supp-4 Supplemental Information 4 Stereo-pair images of *Polonolimulus zaleziankensis* gen. et sp. nov. holotype (latex peel gz4142 of holotype Muz. PGI 1808.II.10) Angle difference between the images is ca. 9 degrees. Scale bars: 10 mm. Images converted to greyscale. Photo credit: Jonatan Audycki.

10.7717/peerj.20950/supp-5 Supplemental Information 5 Examples of Mesozoic xiphosurids included in Austrolimulidae and *Limulitella* (A) *Psammolimulus gottingensis*, GZG.INV.45730a; (B) *Attenborolimulus superspinosus*, PIN 5640/220; (C) *Batracholimulus fuchsbergensis*, SMF VIII 311; (D) *Franconiolimulus pochankei*, SSN 8PG35; (E) *Limulitella bronni*, LIM 68; (F) *Limulitella volgensis*, PIN 4048/7; (G) *Limulitella tejraensis*, ZPAL V.46/101; (H) *Limulitella* cf. *liasokeuperinus*, SNSB-BSPG 1967 XVI 27; (I) *Vaderlimulus tricki*, UCM 140.25; (J) *Tasmaniolimulus patersoni*, UTGD 123979; (K) *Dubbolimulus peetae*, MMF 27693; (L) *Austrolimulus fletcheri*, AM F38274. Scale bars: (D, I, L): 20 mm; (E, G, J): 10 mm; (A, B, F, H, K): 5 mm; (C): 2 mm. Photo credit: (A) Gerhart Hundertmark; (B) Sergey Bagirov; (C) Norbert Hauschke; (F) Constantine Tarásenko; (H) Mike Reich; (I) Allan Lerner; (J) Russell Bicknell; (K) David Barnes; (L) Josh White; (D, E, G) Jonatan Audycki. Images in (A, C, F, H, I, J , K, L) reproduced from Bicknell & Pates (2020) under CC BY 4.0 license; image in (B) reproduced from Bicknell & Shcherbakov (2021) under CC BY 4.0 license.

## Institutional abbreviations

AM F: Australian Museum, Sydney, NSW, Australia
gz: Zoological collections of the Faculty of Biology, University of Warsaw, Warsaw, Poland
GZG INV: Geowissenschaftliches Zentrum der Georg-August-Universität Geowissenschaftliches Museum, Göttingen, Germany
LIM: Staatliches Museum für Naturkunde Stuttgart (Grauvogel collection), Stuttgart, Germany
MAN: Muséum-Aquarium de Nancy, Lorraine, France
MB.A.: Museum für Naturkunde Leibniz-Institut, Berlin, Germany
MGSB: Museo Geológico del Seminario de Barcelona, Barcelona, Spain
MMF: Geological Survey of New South Wales, Londonderry, NSW, Australia
Muz. PGI: Geological Museum, Polish Geological Institute-National Research Institute, Warsaw, Poland
PIN: Orlov Paleontological Museum of the Borissiak Paleontological Institute Russian Academy of Sciences, Moscow, Russia
PMSL: Slovenian Museum of Natural History (Hitij & Žalohar Paleontological Collection), Ljubljana, Slovenia
SMF: Forschungsinstitut Senckenberg, Frankfurt am Main, Germany
SNSB-BSPG: Staatliche Naturwissenschaftliche Sammlungen Bayern Bayerische Staatssammlung für Paläontologie und Geologie, Munich, Germany
SSN: Paläontologisches Museum Nierstein, Nierstein, Rhineland-Palatinate, Germany
UCM: University of Colorado Museum of Natural History, Boulder, CO, USA
UNISTRA: Collections de paléontologie, Jardin des sciences de l’Université de Strasbourg, Strasbourg, France
UTGD: Geology Department, University of Tasmania, Tasmania, Australia
ZPAL: Institute of Paleobiology Polish Academy of Sciences, Warsaw, Poland

## Additional Information and Declarations

### Data Availability

The following information was supplied regarding data availability: The data and code are available in the Supplemental Files. The data is available at OSF: - Audycki, J. (2026, February 3). Additional files for ”A new Triassic austrolimulid from Poland presents insight into xiphosurid evolution and palaeobiogeography at the dawn of the Mesozoic”. https://doi.org/10.17605/OSF.IO/A4M8F
- Audycki, J. (2026, February 3). Geometric morphometric analysis. https://doi.org/10.17605/OSF.IO/ZAQK9
- Audycki, J. (2026, February 3). 3D model of Muz. PGI.1808.II.10. https://doi.org/10.17605/OSF.IO/CN28R
- Audycki, J. (2026, February 3). Additional images of gz4142 (latex peel of holotype Muz. PGI 1808.II.10). https://doi.org/10.17605/OSF.IO/632RU.

### New Species Registration

The following information was supplied regarding the registration of a newly described species: Publication LSID: urn:lsid:zoobank.org:pub:8CE309E7-0B35-493B-89E4-0D16FB7FFDD5 Genus LSID: urn:lsid:zoobank.org:act:9C6EFC6F-D512-4CB2-832C-9D55C2117721 Species LSID: urn:lsid:zoobank.org:act:7A88798F-6FC7-459A-9398-141C22174C57.
