# Supplementary material for: A new Triassic austrolimulid from Poland presents insight into xiphosurid evolution and palaeobiogeography at the dawn of the Mesozoic
Source: PeerJ. 2026 Mar 25;14:e20950. doi: 10.7717/peerj.20950 (PMC13032753; doi:10.7717/peerj.20950)
Supplement: Supplemental Information 1 [file peerj-14-20950-s001.doc]

| **No.** | **Specimen** | **Genus** | **Species** | **Photograph source** | **Source figure details** | **Was the image mirrored for analysis?** |
| --- | --- | --- | --- | --- | --- | --- |
|  | UTGD 123979 | *Tasmaniolimulus* | *T. patersoni* | Bicknell RDC, Smith PM, Brougham T, Bevitt JJ. 2022. An earliest Triassic age for Tasmaniolimulus and comments on synchrotron tomography of Gondwanan horseshoe crabs. *PeerJ* 10:e13326. DOI: 10.7717/peerj.13326. | Fig. 3 A | Yes |
|  | UCM 140.25 | *Vaderlimulus* | *V. tricki* | Bicknell RDC, Pates S. 2020. Pictorial Atlas of Fossil and Extant Horseshoe Crabs, With Focus on Xiphosurida. *Frontiers in Earth Science* 8:98. DOI: 10.3389/feart.2020.00098. | Fig. 25 E | No |
|  | GZG INV 45730 | *Psammolimulus* | *P. gottingensis* | Bicknell RDC, Pates S. 2020. Pictorial Atlas of Fossil and Extant Horseshoe Crabs, With Focus on Xiphosurida. *Frontiers in Earth Science* 8:98. DOI: 10.3389/feart.2020.00098. | Fig. 26 C | Yes |
|  | PIN 5640/220 B[[1]](#footnote-2) | *Attenborolimulus* | *A. superspinosus* | Bicknell RDC, Shcherbakov DE. 2021. New austrolimulid from Russia supports role of Early Triassic horseshoe crabs as opportunistic taxa. *PeerJ* 9:e11709. DOI: 10.7717/peerj.11709. | Fig. 3 A | Yes |
|  | UNISTRA.2015.0.50968 | *Limulitella* | *L. bronni* | Klompmaker AA, Van Eldijk TJB, Winkelhorst H, Reumer JWF. 2023. A non-marine horseshoe crab from the Middle Triassic (Anisian) of the Netherlands*. Netherlands Journal of Geosciences* 102:e1. DOI: 10.1017/njg.2022.16. | Fig. 4 B | Yes |
|  | LIM 68 | *Limulitella* | *L. bronni* | Bicknell RDC, Pates S. 2020. Pictorial Atlas of Fossil and Extant Horseshoe Crabs, With Focus on Xiphosurida. *Frontiers in Earth Science* 8:98. DOI: 10.3389/feart.2020.00098. | Fig. 28 A | Yes |
|  | MMF 27693 | *Dubbolimulus* | *D. peetae* | Bicknell RDC, Smith PM, Brougham T, Bevitt JJ. 2022. An earliest Triassic age for Tasmaniolimulus and comments on synchrotron tomography of Gondwanan horseshoe crabs. *PeerJ* 10:e13326. DOI: 10.7717/peerj.13326. | Fig. 2 A | Yes |
|  | AM F38274 | *Austrolimulus* | *A. fletcheri* | Bicknell RDC, Pates S. 2020. Pictorial Atlas of Fossil and Extant Horseshoe Crabs, With Focus on Xiphosurida. *Frontiers in Earth Science* 8:98. DOI: 10.3389/feart.2020.00098. | Fig. 42 A | No |
|  | MB.A.0207 | ?Limulidae gen. et sp. indet | *“Limulus kieri”* | Bicknell RDC, Pates S. 2020. Pictorial Atlas of Fossil and Extant Horseshoe Crabs, With Focus on Xiphosurida. *Frontiers in Earth Science* 8:98. DOI: 10.3389/feart.2020.00098. | Fig. 31 D | Yes |
|  | PMSL T-993 | *Sloveniolimulus* | *S. rudkini* | Bicknell R, Žalohar J, Miklavc P, Celarc B, Križnar M, Hitij T. 2021. Revisiting horseshoe crab fossils from the Middle Triassic (Anisian) Strelovec Formation Konservat-Lagerstätte of Slovenia. *Palaeontologia Electronica*. DOI: 10.26879/1168. | Fig. 2 A | Yes |
|  | ZPAL V.46/101 | *Limulitella* | *L. tejraensis* | Specimen available directly in Warsaw; new high-resolution photographs produced for the analysis by JA | | Yes |
|  | ZPAL V.46/106 | *Limulitella* | *L. tejraensis* | Specimen available directly in Warsaw; new high-resolution photographs produced for the analysis by JA | | Yes |
|  | ZPAL V.46/120 | *Limulitella* | *L. tejraensis* | Specimen available directly in Warsaw; new high-resolution photographs produced for the analysis by JA | | No |
|  | ZPAL V.46/103p | *Limulitella* | *L. tejraensis* | Specimen available directly in Warsaw; new high-resolution photographs produced for the analysis by JA | | Yes |
|  | MGSB 19195 | *Heterolimulus* | *H. gadeai* | Bicknell RDC, Pates S. 2020. Pictorial Atlas of Fossil and Extant Horseshoe Crabs, With Focus on Xiphosurida. *Frontiers in Earth Science* 8:98. DOI: 10.3389/feart.2020.00098. | Fig. 31 A | Yes |
|  | MGSB M 262 | *Tarracolimulus* | *T. rieki* | Bicknell RDC, Pates S. 2020. Pictorial Atlas of Fossil and Extant Horseshoe Crabs, With Focus on Xiphosurida. *Frontiers in Earth Science* 8:98. DOI: 10.3389/feart.2020.00098. | Fig. 31 C | No |
|  | MAN 8240 | *Keuperlimulus* | *K. vicensis* | Bicknell RDC, Pates S. 2020. Pictorial Atlas of Fossil and Extant Horseshoe Crabs, With Focus on Xiphosurida. *Frontiers in Earth Science* 8:98. DOI: 10.3389/feart.2020.00098. | Fig. 29 A | Yes |
|  | SNSB-BSPG 1967 XVI 27 | *Limulitella* | *L.* cf*. liasokeuperinus* | Bicknell RDC, Pates S. 2020. Pictorial Atlas of Fossil and Extant Horseshoe Crabs, With Focus on Xiphosurida. *Frontiers in Earth Science* 8:98. DOI: 10.3389/feart.2020.00098. | Fig. 30 D | No |
|  | gz4142 (latex peel of the Holotype Muz. PGI 1808.II.10) | *Polonolimulus* gen. nov. | *Polonolimulus zaleziankensis,* gen. et sp. nov. | Specimen available directly in Warsaw; high-resolution photographs produced for the analysis by JA | | Yes |

**Supplemental Table S1**: Specimens used for geometric morphometric analysis

1. Counterpart used as it shows features somewhat better than the part and is not partially cracked [↑](#footnote-ref-2)
