# Supplementary figures and images for: A new Triassic austrolimulid from Poland presents insight into xiphosurid evolution and palaeobiogeography at the dawn of the Mesozoic
Source: PeerJ. 2026 Mar 25;14:e20950. doi: 10.7717/peerj.20950 (PMC13032753; doi:10.7717/peerj.20950)

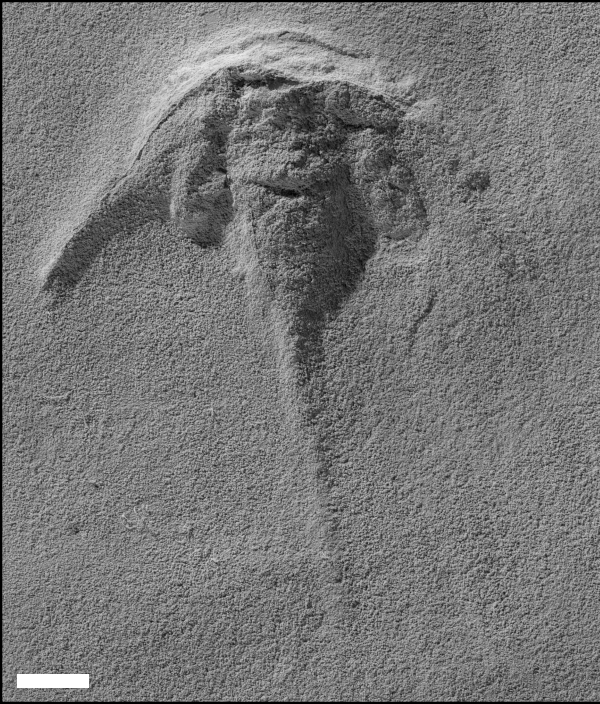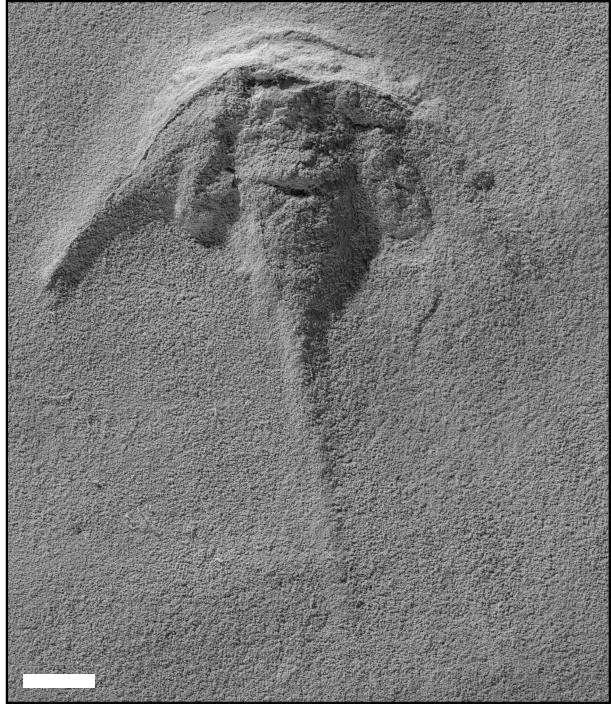

Supplement: Supplemental Information 4 — Angle difference between the images is ca. 9 degrees. Scale bars: 10 mm. Images converted to greyscale. Photo credit: Jonatan Audycki. [file peerj-14-20950-s004.pdf]

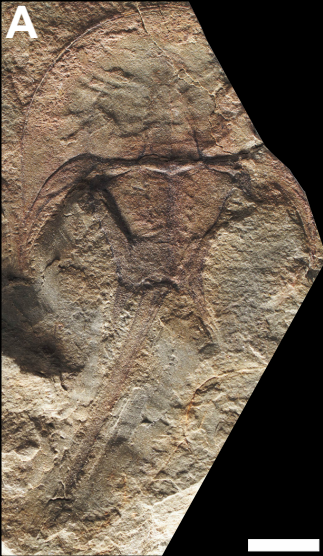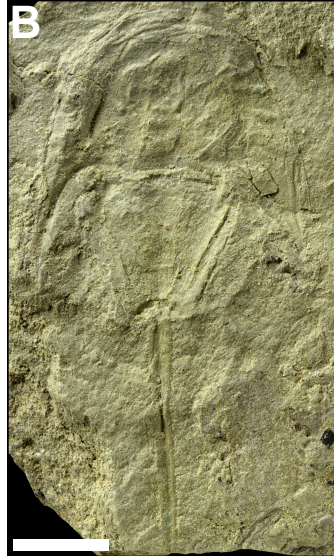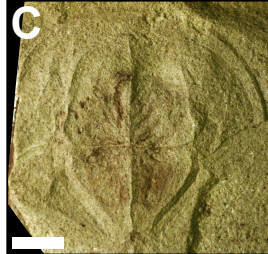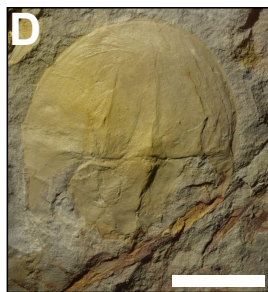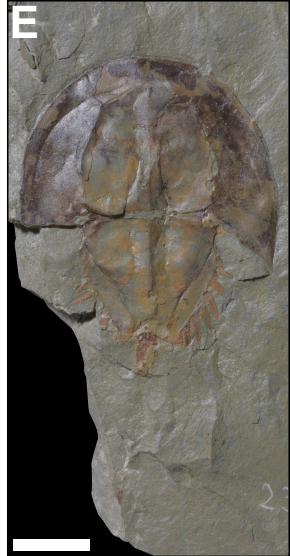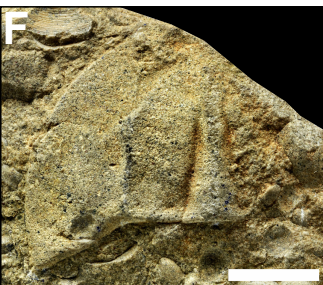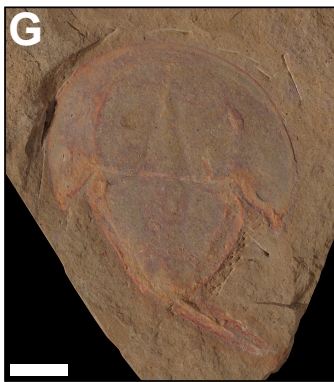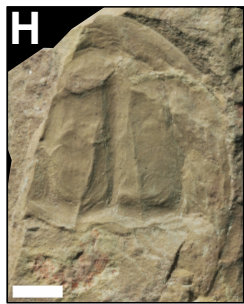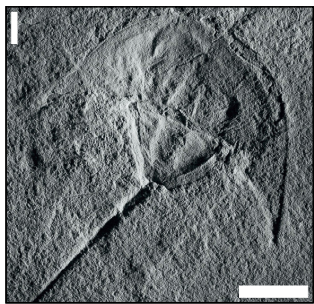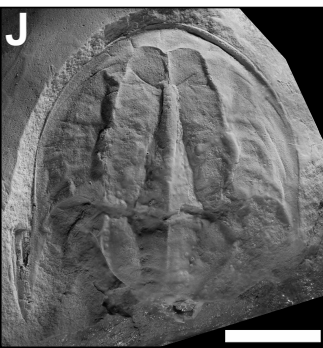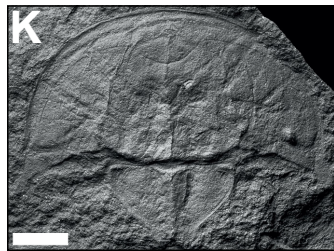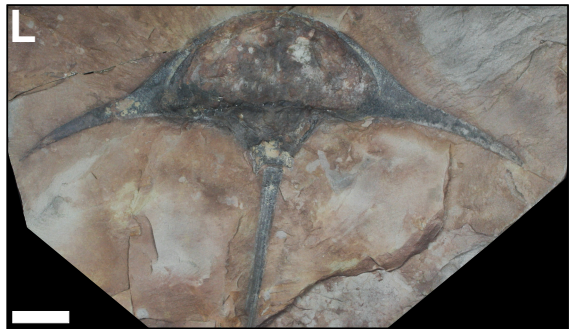

Supplement: Supplemental Information 5 — (A) Psammolimulus gottingensis, GZG.INV.45730a; (B) Attenborolimulus superspinosus, PIN 5640/220; (C) Batracholimulus fuchsbergensis, SMF VIII 311; (D) Franconiolimulus pochankei, SSN 8PG35; (E) Limulitella bronni, LIM 68; (F) Limulitella volgensis, PIN 4048/7; (G) Limulitella tejraensis, ZPAL V.46/101; (H) Limulitella cf. liasokeuperinus, SNSB-BSPG 1967 XVI 27; (I) Vaderlimulus tricki, UCM 140.25; (J) Tasmaniolimulus patersoni, UTGD 123979; (K) Dubbolimulus peetae, MMF 27693; (L) Austrolimulus fletcheri, AM F38274. Scale bars: (D, I, L): 20 mm; (E, G, J): 10 mm; (A, B, F, H, K): 5 mm; (C): 2 mm. Photo credit: (A) Gerhart Hundertmark; (B) Sergey Bagirov; (C) Norbert Hauschke; (F) Constantine Tarásenko; (H) Mike Reich; (I) Allan Lerner; (J) Russell Bicknell; (K) David Barnes; (L) Josh White; (D, E, G) Jonatan Audycki. Images in (A, C, F, H, I, J , K, L) reproduced from Bicknell & Pates (2020) under CC BY 4.0 license; image in (B) reproduced from Bicknell & Shcherbakov (2021) under CC BY 4.0 license. [file peerj-14-20950-s005.pdf]
